# Evaluation of foot functionality in cases of rheumatoid arthritis through the FFI-BR and FHSQ-BR questionnaires: a cross-sectional observational study

**DOI:** 10.1590/1516-3180.2018.0175161118

**Published:** 2018-12-13

**Authors:** Elinah Narumi Inoue, Agnes Patricia de Andrade, Thelma Skare

**Affiliations:** I Undergraduate Medical Student, Faculdade Evangélica do Paraná (FEPAR), Curitiba (PR), Brazil.; II Undergraduate Medical Student, Faculdade Evangélica do Paraná (FEPAR), Curitiba (PR), Brazil.; III MD, PhD. Professor, Department of Rheumatology, Hospital Universitário Evangélico de Curitiba, Curitiba (PR), Brazil.

**Keywords:** Rheumatoid arthritis, Inflammation

## Abstract

**BACKGROUND:**

Rheumatoid arthritis (RA) can affect the feet, thus compromising the patient’s gait and autonomy. In this study, we investigated foot disability in RA patients using the Brazilian versions of the Foot Health Status Questionnaire (FHSQ-BR) and Foot Function Index (FFI-BR).

**DESIGN AND SETTING:**

Cross-sectional, observational study conducted in a tertiary care hospital.

**METHODS:**

Two hundred individuals were studied: 100 with RA and 100 controls. Demographic variables and FFI-BR and FHSQ-BR scores were analyzed. In relation to RA patients, data on medications used and on the following clinical variables were collected: Disease Activity Score-28-ESR; erythrocyte sedimentation rate (ESR), C-reactive protein (CRP) level and rheumatoid factor (RF) level. The groups were compared and the scores and clinical variables were correlated.

**RESULTS:**

RA patients’ scores in the pain, difficulty and disability domains of the FFI-BR questionnaire were worse (P < 0.0001). The FHSQ-BR showed that there were differences between RA patients and controls in relation to the pain and foot function domains: shoes (P < 0.0001), foot health (P < 0.0001), general health (P=0.0002), physical activity (P < 0.0001), social capacity (P = 0.0006) and vigor (P = 0.01). There were correlations between FFI-BR and DAS-28-ESR scores (rho = 0.45), ESR (rho = 0.27) and CRP (rho = 0.24). According to the FHSQ-BR questionnaire, there was a correlation between DAS-28-ESR and worse foot health (rho = 0.29).

**CONCLUSION:**

RA patients’ scores in the foot health assessment questionnaires were worse than those of controls. A correlation between inflammatory activity and worse foot function was found.

## INTRODUCTION

Rheumatoid arthritis (RA) is a chronic, progressive inflammatory disease that can cause limitations and difficulties in activities of daily living (ADLs), due to pain, gait impairment and difficulties with self-care.^1^ Damage to the ankles and feet occurs in 85% to 100% of these patients and contributes greatly to their loss of quality of life.^2^ Typically, foot involvement indicates more aggressive disease and requires more than just pharmacological care.^3^

Studying functional disability of the feet is important because, in addition to causing the abovementioned limitations, this form of disability has been correlated with higher risk of falls due to stiffness, pain, muscle weakness and balance disorders, which increases mortality, health resource utilization and loss of patients’ work capacity.^4^^,^^5^Although RA patients frequently complain about their feet, such complaints are often overlooked by healthcare professionals.

Specific instruments for foot health assessment, including the Brazilian versions of the Foot Health Status Questionnaire (FHSQ-BR) and the Foot Function Index (FFI-BR), measure the day-to-day impact of involvement of the feet in RA. These instruments provide data that help in understanding the repercussions of this involvement and the respective therapeutic indications. This can improve the quality of life of the individuals affected.^3^

## OBJECTIVE

The objectives of the present study were to assess the disability that originated through foot involvement among RA patients, as indicated using the FFI-BR and FHSQ-BR questionnaires, and to evaluate the influence of inflammatory activity on the dysfunction of foot joints.

## METHODS

This was a cross-sectional observational study on individuals diagnosed with RA and controls. It used a convenience sample that included all consecutive RA patients who went for consultations between July 2015 and February 2017 in the same university hospital. They were included in the study in the order of their appointments, according to their willingness to participate in the study.

For subjects to be included in the patient group, they needed to have reached a score of at least six points in the 2010 classification criteria of the American College of Rheumatology (ACR)/European League Against Rheumatism (EULAR).^6^ For subjects to be included in the control group, they needed to be free from chronic inflammatory disease, orthopedic or neuromuscular disease, or any other condition affecting foot health; and to have come for consultation at the dermatology clinic for cosmetic reasons. Controls were matched with RA patients for age, gender and body mass index (BMI).

Approval for this study was granted by the local research ethics committee (date: May 11, 2016; number: 1.560.418). After participants had signed a free and informed consent statement, they were invited to answer two questionnaires, to evaluate the limitations and disabilities caused by foot health problems. The FFI (validated in Portuguese for use in Brazil as FFI-BR) assesses foot functionality; and the FHSQ (validated in Portuguese for use in Brazil as FHSQ-BR) assesses how foot pain impacts on patients’ quality of life. Both of these questionnaires not only evaluate joints but also have already been used to study the foot in general, including skin and nail diseases.^3^

The FFI-BR questionnaire is divided into three domains: disability, difficulty and pain. The responses are given on a numerical scale, from 0 to 10. The total score is obtained for each patient as the arithmetic mean of the three domains and is multiplied by 100 to obtain a percentage. Higher scores mean that foot health is worse and, consequently, so is the individual’s quality of life.^7^

The FHSQ-BR questionnaire also presents three domains. Thefirst assesses foot health in four sections: foot pain, foot function, footwear and general foot health. The second evaluates the patient’s general health, also in four sections: general health, physical activity, sociability and vigor. The third considers the epidemiological characteristics of the interviewees. The FHSQ-BR questionnaire scores were obtained using the Foot Health Status Questionnaire software, version 1.03. The responses were entered in a computer program and a score ranging from 0 to 100 was generated for each domain, such that 0 was the worst condition of the foot, and 100 was the best. The scores reflected the patients’ difficulty in daily activities and the consequences of the disease for the foot.^3^

The RA patients’ medical records were reviewed for epidemiological, clinical and treatment data, namely: age, sex, ethnicity, smoking, weight, height, length of time with the disease, disease activity measured using DAS-28-ESR (Disease Activity Score using 28 joints and erythrocyte sedimentation rate, ESR), ESR, C-reactive protein (CRP), rheumatoid factor (RF), antinuclear antibody (ANA) and medication use. The DAS-28-ESR values were interpreted as follows: < 2.6 = clinical remission; ≤ 3.2 = mild disease activity; <5.2= moderate disease activity; and > 5.2 = severe disease activity.^8^

Data were compiled in frequency and contingency tables. Frequencies were expressed as percentages, while central trend measurements were expressed as means and standard deviations (SD) or as medians and interquartile ranges (IQRs), according to the sample distribution. Comparative analyses on nominal data were done using the chi-square test and Fisher’s test. Numerical data were compared by means of unpaired t and Mann-Whitney tests. The Spearman test was used to make correlations among numerical variables. The significance level was taken to be 5%. The calculations were done with the help of the GraphPad Prism software, version 5.0.

## RESULTS

### a) Description of study sample

In the study period, 200 participants were included, 100 in each group. The pairing of the RA patients and controls and the epidemiological data on these subjects are shown in Table 1.

**Table 1.** Comparison of epidemiological data between rheumatoid arthritis (RA) patients and controls

	RA n = 100	Controls n = 100	P
Female gender (%)	82	82	1.0(*)
Mean age in years (range)	55.2 ± 11.6 (20-76)	53.5 ± 8.98 (36-76)	0.25 (**)
Ethnic background (%)	Caucasians: 69 African descendants: 29 Asians: 2	Caucasians: 69 African descendants: 16 Asians: 3	0.08 (*)
Tobacco exposure (%)	24	19	0.46 (*)
Mean BMI in kg/m² (range)	27.7 ± 4.41 (15.8-43.9)	27.02 ± 4.96 (17.7-46.8)	0.28 (**)

(*) Chi-square test; (**) unpaired t test; BMI = body mass index; n = number.

The median duration of the disease among the sample of RA patients was 9.5 years (range: 1 to 54 years). Among these patients, 56.3% were positive for RF and 32% were positive for ANA.

The median DAS-28-ESR score was 3.13 (IQR = 2.45-4.75), with a range from 0.49 to 8.49. The median ESR was 26mm/h, with a range from one to 105mm; and the median CRP concentration was 6mg/dl, with a range from 0.1 to 54mg/dl.

The treatment profile showed that 18% of the RA patients were treated with antimalarial drugs, 72% with methotrexate, 42% with leflunomide, 31% with anti-TNFα and 1% with other biological drugs.

### b) Comparison of FFI-BR and FHSQ-BR results between RA patients and controls

The comparison between the scores of RA patients and controls, from the FFI-BR and FHSQ-BR instruments, is shown in Table2. This table shows that the RA patients presented worse foot function in all the domains studied, in both questionnaires.

**Table 2.** Scores from the Brazilian versions of the Foot Function Index (FFI-BR) and Foot Health Status Questionnaire (FHSQ-BR) compared between rheumatoid arthritis (RA) patients and controls

	RA (*) n = 100	Control (*) n = 100	P (Mann-Whitney test)
FFI-BR			
Median incapacity	19.0 (0-37.5)	0 (0-0)	< 0.0001
Median difficulty	53.3 (24.4-73.3)	8.33 (0-22.2)	< 0.0001
Median pain	51.4 (28.5-71.4)	15.0 (1.78-28.5)	< 0.0001
FHSQ-BR			
Foot pain	54.3 (18.75-78.13)	79 (54.0-97.0)	< 0.0001
Foot function	68.7 (37.5-87.5)	94 (75-100)	< 0.0001
Shoes	25 (0-58.3)	50 (25.0-75.00)	< 0.0001
General foot health	25 (18.75-60)	60 (25.0-85.0)	< 0.0001
General health	50 (30.0-70.0)	60 (50.0-90.0)	0.0002
Physical activity	50 (27.78-66.67)	83 (67-94)	< 0.0001
Social capacity	75 (37.5-100)	88 (75-100)	0.0006
Vigor	50 (25-75)	62 (38-75)	0.01

(*) All values are stated as medians and interquartile ranges.

### c) Correlation between foot function and RA variables

The correlations relating to both questionnaires are shown in Table 3. These showed that all the domains of the FFI-BR correlated with the disease activity measured using DAS-28-ESR. Thiscorrelation was also shown for all FHSQ-BR domains except for the patient’s social capacity and useof shoes.

**Table 3.** Correlation between scores from the Brazilian versions of the Foot Function Index (FFI-BR) and Foot Health Status Questionnaire (FHSQ-BR) and the inflammatory disease activity measured from the Disease Activity Score using 28 joints and erythrocyte sedimentation rate (DAS-28-ESR)

	Rho (Spearman)	95% CI	P
FFI-BR			
Total score	0.45	0.27-0.60	< 0.0001
Median incapacity	0.46	0.28-0.61	< 0.0001
Median difficulty	0.42	0.23-0.57	< 0.0001
Median pain	0.37	0.17-0.53	0.0002
FHSQ			
Foot pain	-0.40	-0.5 to -0.21	< 0.0001
Foot function	-0.34	-0.51 to -0.15	0.0005
Shoes	-0.19	-0.38 to +0.01	0.06
General foot health	-0.26	-0.44 to -0.05	0.009
General health	-0.29	-0.47 to -0.09	0.03
Physical activity	-0.44	-0.60 to -0.26	< 0.0001
Social capacity	-0.15	-0.35 to +0.005	0.13
Vigor	-0.33	-0.50 to -0.13	0.001

CI = confidence interval.

It was also found that the total score of the FFI-BR questionnaire did not have any association with body mass index (BMI) (P= 0.17), presence of RF (P = 0.49) or presence of ANA (P = 0.30).

There was no association between the domains of the FHSQ-BR and the presence of RF and ANA (P = 0.49 and 0.30 respectively). BMI showed a modest correlation with foot pain (Spearman rho=-0.24; 95% CI = -0.45 to -0.04; P = 0.01), but this was unrelated to the other domains studied, for which P was non-significant in all cases.

## DISCUSSION

In this study, foot dysfunction evaluated using the FFI-BR questionnaire was higher in the RA group than in the control group. The FFI-BR questionnaire evaluates simple activities such as walking around the house and on uneven ground, standing on tiptoes and getting up from a chair. It was observed that the RA patients reported difficulties that were almost seven times greater than those of the controls, with median scores of 53.3 versus 8.3, respectively. Reported foot pain, as assessed using the FFI-BR and including pain in the morning, at the end of the day or when standing, was greater among the patients than among the controls, with median scores of 51.4 versus 15, respectively. Dysfunction and pain generate disability, which in this study was also higher among the patients than among the controls. Footdysfunction measured using the FFI-BR shows individuals’ limitations regarding going out from their homes, along with their need to use orthopedic devices and their loss of independence in relation to simple day-to-day activities.

A similar trend was found regarding foot function measured using the FHSQ-BR questionnaire. Although impaired, the function of our patients’ feet proved to be better than was seen in Ferreira’s sample,^9^ among patients from a reference outpatient clinic in São Paulo. In that study, the score was 48, while in the current study it was 68.7.^9^ Use of new drugs for treating RA and more aggressive treatment strategies that enable better control over the inflammatory activity of RA may explain this difference.

Impairment of RA patients’ motor capacity can compromise their lifestyle, through mechanical difficulties and insecurity, thereby depriving them of their daily activities, social living and ability to access different places.^10^ Additionally, this impairment leads to increasing dependence on family support and consequent frustration and anger, which promote social isolation, emotional instability and depression.^11^ Anxiety and depression are associated with reduced physical activity and sedentary lifestyle, and thus their presence ends up worsening these individuals’ general health in the long run.^12^^,^^13^ This was reflected, in our study, in the social capacity assessed through the FHSQ-BR questionnaire, which showed worse scores in the group of patients than among the controls. Incomparison with Ferreira’s sample, our patients had better results regarding sociability, thus showing that those with worse foot health also had worse social performance.^9^

In evaluating the shoes domain of the FHSQ-BR questionnaire, RA patients reported that they had twice as much difficulty in finding adequate shoes, compared with the control group, and that they could not use all types of shoes. The complaints about shoes were even worse in Ferreira’s sample, in which the patients scored 5.8, i.e. around five times lower than in the present study.^9^ This difference may have been related to fewer footwear options at that time or to less knowledge about the subject.

Some of the difficulties found by RA patients regarding shoes probably resulted from the fact that the shoe industry focuses on esthetics rather than comfort and functionality, thus leaving the population with special needs without any choice. The alternative is to search for orthopedic shoes and insoles that are customized for patients, even though their cost is much higher than that of ordinary shoes. Moreover, the greatest challenge in relation to adherence to orthopedic shoes is patients’ own resistance to them, due to their unattractive appearance.^14^ Many RA patients find it difficult to tie shoe laces because of hand deformities: for these individuals, footwear with Velcro fastenings or elastic openings are options of greater interest. Non-slip soles can also help in fall prevention. Inaddition, feet also have esthetic importance; deformities may contribute towards greater personal dissatisfaction, especially among women, who are more affected by this rheumatic disease.^11^

It was evident from both questionnaires in our study that disease activity, as evaluated using DAS-28-ESR, affected foot health. Thus, strict control over disease activity is a way to avoid loss of foot function, and this needs to be implemented from the time of the initial diagnosis.

Body mass index (BMI) usually affects foot function. An excess of mechanical loading on the knee, ankle and foot joints worsens local pain.^15^Additionally, obese patients’ response to treatment is worse, with lower likelihood of remission from the disease.^16^ Therefore, stimulating weight loss among obese or overweight patients contributes towards their physical and emotional wellbeing and demonstrates care for the patient’s health as a whole.^16^ Interestingly, in our study, only the pain assessed according to the FHSQ-BR questionnaire correlated with the BMI value. One possible hypothesis for explaining this is that the degree of inflammation had such an important influence that it caused foot dysfunction even in individuals with low BMI.

This study had limitations and may not have accurately represented patients with RA in general because it was conducted in an outpatient clinic at a tertiary-care hospital, where patients have access to specialized and effective treatment. Greater severity of disease due to uncontrolled disease activity may be seen outside this sample. The Health Assessment Questionnaire (HAQ), an instrument that is used to study functional status in rheumatic diseases,^17^ was not used in the present analysis and this was therefore a limitation of this study. Another limitation was the subjectivity of the questionnaires, since ascertaining the dimensions of patients’ pain depended directly on the patients’ understanding ofthe questions and on their capacity to understand scales.

Negligence of the impact of foot health on patients’ quality of life occurs because feet dysfunction is often interpreted as being natural to the aging process and to the disease. This leads to omission of treatment and delays to it, in this segment of the body.^13^

A holistic approach with multidisciplinary treatment, social support and public health policies combating architectural barriers may help RA patients to ensure their autonomy and ability to function as citizens through ensuring their mobility and independence.

## CONCLUSION

We concluded that patients with RA had worse scores in the FFI-BR and FHSQ-BR foot health questionnaires, compared with individuals who did not have the disease. Inflammatory activity is a major determinant of the health of this body segment and it needs to be brought under control in order to improve foot health.
